# Exploring robustness of hybrid membranes under high hydrostatic pressure and temperature

**DOI:** 10.3389/fmicb.2024.1470844

**Published:** 2024-11-14

**Authors:** Anandi Tamby, Diana X. Sahonero-Canavesi, Laura Villanueva

**Affiliations:** ^1^Department of Marine Microbiology and Biogeochemistry (MMB), NIOZ Royal Netherlands Institute for Sea Research, Den Burg, Netherlands; ^2^Department of Biology, Faculty of Sciences, Utrecht University, Utrecht, Netherlands

**Keywords:** lipid divide, high hydrostatic pressure, membrane adaptation, membrane lipids, bacteria, cell morphology

## Abstract

Bacterial membranes are typically composed of ester-bonded fatty acid (FA), while archaeal membranes consist of ether-bonded isoprenoids, differentiation referred to as the ‘lipid divide’. Some exceptions to this rule are bacteria harboring ether-bonded membrane lipids. Previous research engineered the bacterium *Escherichia coli* to synthesize archaeal isoprenoid-based ether-bonded lipids together with the bacterial FA ester-linked lipids, showing that heterochiral membranes are stable and more robust to temperature, cold shock, and solvents. However, the impact of ether-bonded lipids, either bacterial or archaeal, on membrane robustness, remains unclear. Here, we investigated the robustness, as survival after shock, of *E. coli* synthesizing either archaeal or bacterial ether-bonded membrane lipids, under high temperature and/or high hydrostatic pressure (HHP). Our findings reveal *E. coli* with bacterial ether-bonded lipids is more robust under HHP and high temperature. On the contrary, the presence of archaeal ether-bonded membrane lipids in *E. coli* does not affect the robustness under HHP nor high temperature under the tested conditions. We observed morphological changes induced by the shock treatments including reduced length under high temperature or HHP, and the presence of elongated cells after a shock of HHP and high temperature combined, suggesting the combined treatments impaired cell division. Our results contribute to a deeper understanding of membrane adaptation to extreme environmental conditions and highlight the significance of HHP as a key parameter to investigate the differentiation of membranes during the lipid divide.

## 1 Introduction

The cytoplasmic membrane is an essential component of the cell, ensuring the transport of nutrients within the cell while protecting biomolecules such as DNA or cytoplasmic proteins (Yeagle, 1989). Bacterial and eukaryotic cell membranes typically consist of lipids with fatty acids bound to glycerol-3-phosphate through an ester bond, while archaeal lipids differ by harboring isoprenoid chains bound to glycerol-1-phosphate through ether bonds (Siliakus et al., 2017). Bacterial lipids are organized into bilayers while archaeal lipids can either be organized as monolayers of membrane-spanning lipids (MSL) or so-called glycerol dialkyl glycerol tetraethers (GDGTs), or as bilayers of glycerol diethers [archaeol; (Schouten et al., 2013)]. Those drastic differences between the membrane lipids of Archaea and Bacteria/Eukarya are referred to as the ‘lipid divide’. Nevertheless, how this process took place, and the evolutionary and/or adaptative force leading to the separation of the two membrane types is still under debate (Koga, 2011; Villanueva et al., 2017). One of the hypotheses is that cell membranes went through a stage of hybrid membrane containing both archaeal and bacterial-like membrane lipids leading to the ‘lipid divide’ (Koga, 2011). A study by Caforio et al. (2018) engineered the bacterium *Escherichia coli* to synthesize up to 30% of unsaturated archaeol-type (C_20_ isoprenoids in a bilayer) archaeal lipids, proving that membranes with mixed lipids (i.e., archaeal and bacterial-like) can be stable and thus further supporting a ‘lipid divide’ stage in the evolution of cell differentiation. An exception to the ‘lipid divide’ is the presence of ether-linked fatty acid-based membrane lipids in some bacterial groups, sometimes found as MSLs.

Recently, the key gene-coding enzymes involved in the formation of the bacterial MSLs by the condensation reaction of the tail of two fatty acids (i.e., membrane-spanning lipid synthase coding gene, *mss*), and of the synthesis of alkyl ether bonds (i.e., glycerol ester reductase coding gene, *ger,* a homolog of the plasmalogen synthase PlsA involved in the formation of alkenyl ethers) have been constrained and their function confirmed by heterologous gene expression in *E. coli* (Sahonero-Canavesi et al., 2022a). Several bacterial groups have been predicted to harbor the capacity to synthesize MSL, ether bonds, or both based on the presence of those coding genes in their genomes (Sahonero-Canavesi et al., 2022a). However, the presence of these genes is quite widespread without a clear correlation to bacterial groups that can withstand specific environmental conditions. Thus, it is still unclear whether the presence of these lipids might provide an adaptative advantage under different environmental pressures. Overall, it seems that the capacity to synthesize mixed archaeal-bacterial membrane lipids is more widespread than originally thought, but the physiological or environmental advantage of this characteristic is unknown.

Several environmental factors have been tested to assess their effect on the stability of microbial membranes and on lipid vesicles to mimic different origin of life scenarios. For example, ether bonds in bacterial lipids have been suggested to improve cell resistance against environmental stresses (Koga and Morii, 2005; Lehmann et al., 2023). The reason for the presence of these archaeal like features (i.e., monolayers and ether bonds) in some Bacteria remains unclear but it has been suggested that these characteristics could be contributing to the stability of the membrane at high temperature or low pH, as an increase of these lipids has been observed under those conditions (Valentine, 2007; Sahonero-Canavesi et al., 2022b; Halamka et al., 2023).

Higher temperatures favor microorganisms with archaeal membranes or those with membrane spanning bacterial ether lipids (Jain et al., 2014). In this regard, environmental settings with high temperatures very often coincide with high hydrostatic pressure (HHP), as common factors co-occurring in the deep sea. These environments with temperature and/or HHP have been commonly thought to be potential scenarios for the events leading to the origin of life and of the evolution of cell forms (Daniel et al., 2006; Xiao et al., 2021). The role of HHP in modifying membrane characteristics, including lipids, has been previously studied and involves an increase of plasmalogen and unsaturated and branched fatty acids in bacterial phospholipids with increasing HHP to maintain the homeostasis of the membrane (Bartlett, 2002; Tamby et al., 2023; Winnikoff et al., 2024). However, this adaptation is distinct from the mechanisms involved in surviving high pressure extremes. Yet, little is known of how HHP, and its combination with high temperature, can affect cell membranes containing lipids with archaeal and bacterial-like features, which would be key information to further understand the process of ‘lipid divide’ through an intermediate stage of membranes with mixed characteristics.

Here, we evaluated the individual or combined effect of two key environmental factors, which have likely played an important role during the evolution of cell membranes (i.e., high temperature and HHP), on the robustness and cell shape of a model microorganism (i.e., *E. coli*) synthesizing either mixed bacterial/archaeal (archaeol) membranes or mixed membranes containing bacterial ester-bonded and ether-bonded phospholipids. Results indicate that the presence of ether-bonds increases the robustness at high temperatures, especially so after a high-pressure shock, and that mixed bacterial/archaeal membranes display comparable robustness to their bacterial counterpart. We also observed that a combined shock of HHP and temperature induced an elongation in cells, which is not observed when high temperature and HHP shocks are applied separately, thus showcasing that HHP and temperature are crucial parameters to consider together for the study of membrane adaptation.

## 2 Materials and methods

### 2.1 Strain description and growth under different conditions

*Escherichia coli strain synthesizing bacterial membrane lipids with ether bonds.* Genes from *Thermotoga maritima* encoding for the membrane-spanning lipid synthase (*mss*) and the glycerol ester reductase (*ger*) were identified prior to this study (Sahonero-Canavesi et al., 2022a). Co-expressions of those genes from *T. maritima* in *E. coli* was performed by transforming the *ger*-containing expression plasmid (pLS12) into the *E. coli* BL21 DE3 containing the *mss*-containing expression plasmids (pLS29) (see Supplementary Tables S1A,B; Sahonero-Canavesi et al., 2022a). Cells were grown anaerobically and induced with a final concentration of 5 mM isopropyl-*β*-D-thiogalactoside (IPTG). Cells were grown to the exponential phase in 2xYT media (see below for details) and harvested by centrifugation and pellets washed with 0.85% NaCl solution. Total lipid extraction of the cell pellets was performed as indicated in Sahonero-Canavesi et al. (2022a). The membrane composition of the cell pellets was then analyzed by gas chromatography mass spectrometry (GC–MS) as indicated in Sahonero-Canavesi et al. (2022a). The percentage of ether-bonded lipids [i.e., C16:0 monoglycerol ether MGE; (see Sahonero-Canavesi et al., 2022a)] was estimated to be less than 0.5% of all fatty acids detected (due to coelution of the C16:0 MGE with a non-identified compound, it was not possible to quantify the C16:0 MGE accurately). This *E. coli* strain is referred to a*s ‘E. coli with bacterial ether-based membrane lipids’*. To confirm our experimental results with this strain, a negative control consisting of an *E. coli* host with empty expression vectors was used (see Supplementary Table S1B).

*E. coli strain synthesizing archaeal membrane lipids with ether bonds*. Here, an *E. coli* strain similar to the one reported by Caforio et al. (2018), modified to produce archaeal membrane lipids, but with the genes coding for the GGGP and DGGGP synthases derived from an uncultured bacterial strain of *Candidatus* Cloacimonetes (Villanueva et al., 2020), was used. *E. coli* C43 strain was transformed with the pMS148 plasmid harboring the *crtE* gene for enhanced isoprenoid production and ether bonds was used as negative control (Table 1). The *E. coli* C43 freshly transformed with pMS148 and pABW3 (carrying the GGGPS and DGGGP synthase coding genes of *Candidatus* Cloacimonetes, Villanueva et al., 2020) (see Table 1), was used to test the effect of the production of archaeal ether-based membrane lipids. Cells were grown until the exponential phase. After 16 h in Mg-Terrific broth (see below for details) cells were harvested by centrifugation and the pellets washed with 0.85% NaCl solution.

**Table 1:** Summary of the genetic composition of the two strains under study, as well as their membrane features.

Strain	Plasmids	Name	Genes	Hybrid features	References
*Escherichia coli* BL21	pLS29	*E. coli* with bacterial ether-based membrane lipids	*mss* from *Thermotoga maritima*	Ether bonds binding FA to the headgroup	Sahonero-Canavesi et al. (2022a)
	pLS12		*ger* from *Thermotoga maritima*		
*Escherichia coli* C43	pMS148	*E. coli* with archaeal diethers	*crtE* from *Pantoea ananatis araM*	Isoprenoid attached to an AG (archaetidylglycerol) or AE (archaetidylethanolamine) through ether bonds	Caforio et al. (2018) and Villanueva et al. (2020)
	pABW3		GGGPS and DGGGPS from *Cloacimonetes*		

### 2.2 Media preparation

2xYT media was prepared anaerobically using tryptone (16 g/L), yeast extract (10 g/L) and NaCl (5 g/L). 1 L of media was then aliquoted in 35 mL in serum bottles and sealed with butyl stoppers. The headspace was then flushed with N_2_ for 10 min to make the medium anoxic, after which the serum bottles were autoclaved at 121°C for 20 min. After sterilization, each bottle was supplemented with streptomycin (100 μg/mL), kanamycin (50 μg/mL) and stock solutions of NaNO_3_ (2 M) and 3-(N-morpholino) propane sulfonic acid (MOPS, 2 M) to a final concentration of 20 mM and kept at 37°C until inoculation.

Mg-Terrific broth was prepared aerobically using tryptone (12 g/L), yeast extract (24 g/L), glycerol (4 g/L), KH₂PO₄ (2.3 g/L), MgSO_4_ x 7H_2_O (0.4 g/L), and K₂HPO₄ (12.5 g/L). 1 L of media was sterilized by autoclaving at 121°C for 20 min. After sterilization, the media was aliquoted under sterile conditions in 35 mL in falcon tubes and kept overnight at 37°*C. prior* to the inoculation, each tube was supplemented with antibiotic for a final concentration of 50 μg/mL for kanamycin and 100 μg/mL for ampicillin.

### 2.3 Cultivation method

*Escherichia coli* with bacterial ether-based membrane lipids and its control strain (Table 1) were inoculated in (1,20) anaerobic 2xYT media supplemented with kanamycin (50 μg/mL) and streptomycin (100 μg/mL), grown overnight to an optical density 600 nm (OD_600_) of 0.4 at 37°C, then inoculated in triplicate (dilution 1:100) in anaerobic 2xYT medium supplemented with antibiotics. Both freshly inoculated cultures were grown to an OD_600_ of 0.3 and then induced with IPTG to a final concentration of 5 mM for 24 h at 37°C. After 24 h, the grown cultures were shocked for 15 min with either HHP (50 MPa), high temperature (47°C) or a combination of both. In the case of HHP shock, the cultures were transferred to a high-pressure cultivation assembly previously described by Yadav et al. (2020). To achieve the temperature of incubation (37°C or 47°C), the assemblies were kept in an incubator for 45 min at the target temperature for the equipment to achieve the incubation temperature. The cultures were then transferred to the assemblies and then placed back in the incubator to proceed with the experiment. They were kept there for 15 min and then taken out to cool down for one hour until reaching ambient temperature prior to decompression. Samples of 1 mL were collected for OD_600_ measurements, and 1 mL was collected for microscopy. Further, robustness was assessed by serial dilution to a factor of 10^−6^ (with 2xYT media for *E. coli* producing ether-based lipids) or of 10^−12^ (with Mg-Terrific broth for *E. coli* producing archaeal lipids) and subsequent plating on agar plates of 25 mL medium containing antibiotics. The remaining biomass was harvested by centrifuge (5,000 rpm, 4°C), and the pellets were washed with 0.85% NaCl solution and frozen at −80°C prior to lipid analysis.

*E. coli* C43 pMS148 clones were freshly transformed with pABW3 plasmid (Table 1) through heat-shock (42°C, 20 s), and plated on LB plates supplemented with kanamycin and ampicillin and grown at 37°C. After 24 h, three individual clones were inoculated in liquid LB supplemented with antibiotic and grown overnight at 37°C, 250 rpm. The culture was diluted 1:20 and grown until OD600nm reached 0.4 to 0.6. It was then diluted in Mg-Terrific broth to an OD600nm of 0.01 and induced with IPTG to a final concentration of 5 mM for 16 h. The same shock and sample treatment was then performed.

Comparisons between colony forming units (CFU) count 24 h after dilution were done on GraphPad Prism (10.1.2). For absolute count of CFU, 2way ANOVA test were performed, while relative count was done with one-way ANOVA test, using unshocked culture as reference. For both, significance level was set at *p* < 0.05.

### 2.4 Lipid analysis

Cells pellets obtained as described above were extracted as follows. Core lipids (without polar head groups) from ‘*E. coli* containing bacterial ether-based membrane’ were extracted from freeze-dried pellets and analyzed as previously described (Sahonero-Canavesi et al., 2022a). Core lipids were identified based on literature data and library mass spectra.

Intact polar lipids (containing polar head groups) from ‘*E. coli* containing archaeal membrane lipids with ether bonds’ were extracted twice in an ultrasonic bath for 10 min in solution of methanol:dichloromethane:phosphate (2:1:0.8, v:v). The supernatants were then combined and phase-separated by adding dichloromethane and phosphate buffer to a final ratio of 1:1:0.9 (v:v). The organic phase was collected, and the aqueous phase was extracted twice with dichloromethane. The biomass residue was then re-extracted following the same procedure but with a mixture of MeOH:DCM:TCA (aqueous trichloroacetic acid solution; pH 3; 2:1:0.8, v:v). Finally, all organic extracts were combined and dried under N_2_. For analysis the extracts were redissolved in MeOH:DCM (9:1, v:v) which contained an internal standard, a deuterated betaine lipid (1,2-dipalmitoyl-*sn*-glycero-3-O-4′-[N,N,N-trimethyl (d9)]-homoserine; Avanti Lipids), before filtration through 0.45 mm pore size regenerated cellulose syringe filters (4 mm diameter; Grace Alltech).

The Bligh-Dyer (BD) extracts were analyzed by ultra-high performance liquid chromatography coupled with high-resolution mass spectrometry (UHPLC-HRMS) according to Bale et al. (2021). To conduct IPL analysis, we used an Agilent 1,290 Infinity I UHPLC equipped with thermostatted autoinjector and column oven, coupled to a Q Exactive Orbitrap MS with an Ion Max source and heated electrospray ionization (HESI) probe (Thermo Fisher Scientific). Separation was achieved on an Acquity BEH C18 column (Waters, 2.1 × 150 mm, 1.7 μm) maintained at 30°C. We used an eluent composition of (A) methanol/water/formic acid/14.8 M NH_3_aq [85:15:0.12:0.04 (v:v)] and (B) isopropyl alcohol/methanol/formic acid/14.8 M NH_3_aq [50:50:0.12:0.04 (v:v)]. The elution program was: 95% A for 3 min, followed by a linear gradient to 40% A at 12 min and then to 0% A at 50 min, this was maintained until 80 min. The flow rate was 0.2 mL.min^−1^. Positive ion HESI settings were: capillary temperature, 300°C; sheath gas (N_2_) pressure, 40 arbitrary units (AU); auxiliary gas (N_2_) pressure, 10 AU; spray voltage, 4.5 kV; probe heater temperature, 50°C; S-lens 70 V. Lipids were detected using a mass range of m/z 350–2000 and MS2 spectra were obtained via data-dependent acquisition, where the top ten abundant ions per MS1 scan were selected for fragmentation. During analysis, dynamic exclusion was used to temporarily exclude masses (for 6 s) to allow selection of less abundant ions for MS/MS. Stepped normalized collision energy (NCE) of 15, 22.5 and 30 was used for fragmentation. The amount of AG was determined by integration of the peak area of the summed mass chromatograms of the protonated, ammoniated and sodiated ion ([M + H]+, [M + NH_4_]+, [M + Na]+) within 3 parts per million (ppm) relative mass tolerance. This was then compared to the peak area of the total base peak chromatogram.

### 2.5 Cell visualization by microscopy

1 mL of culture was sampled and centrifuged right after shock treatment (5 min, 14,000 rpm). After discarding the supernatant, the pellets were preserved overnight at 4°C until staining. The cells were then stained for 3 min at room temperature with a 1x staining buffer containing the membrane dye FM4-64 (1 μg/mL), membrane permeable DNA dye 4′,6-diamidino-2-phenylindole dihydrochloride (DAPI) (5 μg/mL), and membrane impermeable DNA dye SYTOX green (0.5 μM) and disposed on an 0.5% agarose pad. The cells were visualized using an Axio Imager.M2 equipped with Axiocam 705 color and Axiocam 705 mono with fluorescent filters 02 (G 365, FT 395, LP 420) for DAPI, 09 (BP 450–490, FT 510, LP 515) and 00 (BP 530–585, FT 600, LP 615) for FM4-64 and SYTOX green.

Filter sets from Zeiss were used to capture the image (02 for DAPI and 09 for SYTOX green and FM4-64). Images stained with DAPI, SYTOX and FM4-64 were captured independently (Sahonero-Canavesi et al., 2022b) with a depth of field (DOF) between 0.84 μm and 1.13 μm. The objectives used were Plan-Neofluar 100x/1.30 Oil Ph3. The quantitative analysis was performed on 100 cells with ImageJ (version 1.46r) through the MicrobeJ (version 5.13p) plug-in according to Sahonero-Canavesi et al. (2022b). For this analysis, captured stained with DAPI (nucleic acid stain, 5 μg/mL) was used. Each experimental condition was analyzed in triplicate through this method. Statistical analysis was performed using GraphPad Prism (10.1.2) software. Differences between conditions were assessed using ordinary one-way ANOVA test, with a significance level set at *p* < 0.05.

## 3 Results and discussion

### 3.1 Lipid composition of the *E. coli* strains under study

‘*E. coli containing bacterial ether-based lipids’*. The enzyme responsible for producing membrane-spanning lipids (Mss) and the enzyme converting an ester bond to an ether bond (Ger) were previously identified by Sahonero-Canavesi et al. (2022a), and individually confirmed by heterologous gene-expression to produce the membrane-spanning or ether lipids products. With the aim to synthesize membrane-spanning lipids with ether bonds in *E. coli*’s membrane, like those naturally occurring in *T. maritima*, we transformed the plasmid containing the Ger coding gene from *T. maritima* in a *E. coli* strain (*E. coli* BL21 DE3 containing the Mss coding gene from *T. maritima*). We hypothesized that the co-expression of both genes would produce membrane-spanning ether-bonded lipids. Surprisingly, we only detected lipids containing ether-bonds in the *sn*-1 position (see Supplementary Figure S1), but not membrane-spanning ether bonded-containing lipids. This could be due to poor Mss protein expression due to the co-induction of both genes, that the produced enzyme was inactive after expression (e.g., by confinement in inclusion bodies), or toxic protein expression due to excess of induction, which would explain the lack of membrane-spanning lipids. Alternatively, the combined expression of the *mss* and *ger* genes might have caused cellular stress for the *E. coli* strain harboring both plasmids. Regardless, this strain was still producing ether-bonded fatty acid membrane lipids in its membrane (estimated as less than 1% of total fatty acids detected). We proceeded with the evaluation of the effect of these ether-bonded lipids on the strain’s robustness. Consequently, we refer to this strain from this point on as ‘*E. coli* containing bacterial ether-based lipids’, which we used in the subsequent tests to determine the effect of the presence of bacterial ether-bonds in the robustness of this *E. coli* strain under different conditions.

*‘E. coli containing archaeal membrane lipids with ether bonds’*. The lipid composition of the strain *E. coli* C43 transformed with pMS148 and pABW3 (with GGGPS and DGGGP) synthase coding genes of *Candidatus* Cloacimonetes, (Villanueva et al., 2020) (see material and methods for details) was reported by Villanueva et al. (2020), showing the production of archaeal ether-bonded membrane lipids. Here, we confirmed again the production of these lipids by intact polar lipid analysis as described in Supplementary Figure S2, prior to the use of this strain for the subsequent steps to determine its robustness under different conditions and estimated its relative abundance 0.05% ± 0.01. The tetraether synthase (Tes), involved in the linking of two archaeol diethers molecules leading to glycerol dialkyl tetraethers, GDGTs, was not included in this expression system, therefore only archaeol-like diether molecules are expected.

### 3.2 Experimental set-up and general effect on colony morphology

To assess the effect of the presence of ether bonded-membrane lipids in the robustness (survival after shock) of *E.coli*, the strains confirmed to harbor either bacterial or archaeal ether-bonded lipids in their membranes were grown until mid-exponential phase then shocked with either high temperature (10°C above the optimal; i.e., 47°C), HHP (50 MPa) for 1 h, or a combination of HHP (50 MPa) for 1 h and high temperature (47°C) for 15 min before evaluating their robustness to these shocks. Robustness was assessed by estimating survival based on determining CFU able to grow on a media plate after a 24 h incubation following the applied shock, as well as the percentage of the CFU grown after shock relative to those of the control *E. coli* cells (not producing ether-bonded lipids) after the shock. *E. coli* with bacterial ether-bonded membrane lipids, the effect of a concomitant short shock between high temperature (47°C) and HHP (50 MPa) for 15 min was assessed.

All *E. coli* strains grown after the applied shock on solid agar plates were transparent and smaller with respect to the unshocked controls, where colonies were whiter and thicker, which suggests these changes in colony morphology are induced by the applied stress (Marietou et al., 2014), or alternatively due to the handling of the culture as a physiological response. Reduction of colony size upon stress has been observed in former studies and linked to starvation in prolonged stationary phase, as well as under HHP, highlighting the effect of the applied stress factors on the growth of the cells (Zapparoli, 2004; Marietou et al., 2014).

### 3.3 Effect of the presence of ether-bonded bacterial membrane lipids on robustness

**Figure 1:**
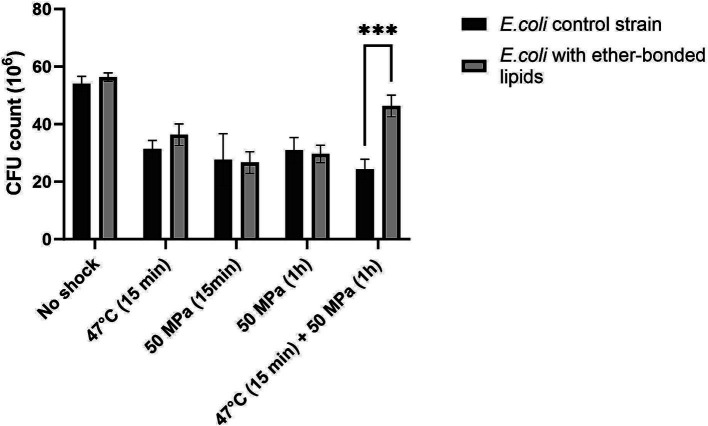
Colony forming units (CFU) absolute count in *E. coli* strain not synthesizing ether bonded membrane lipids, and in *E. coli* producing bacterial ether-bonded membrane lipids after different shock HHP and/or high temperature treatments. Experiments were performed in triplicate. Error bars indicate ± standard deviation (SD). *** Means strongly significant (*p* ≤ 0.001).

**Figure 2:**
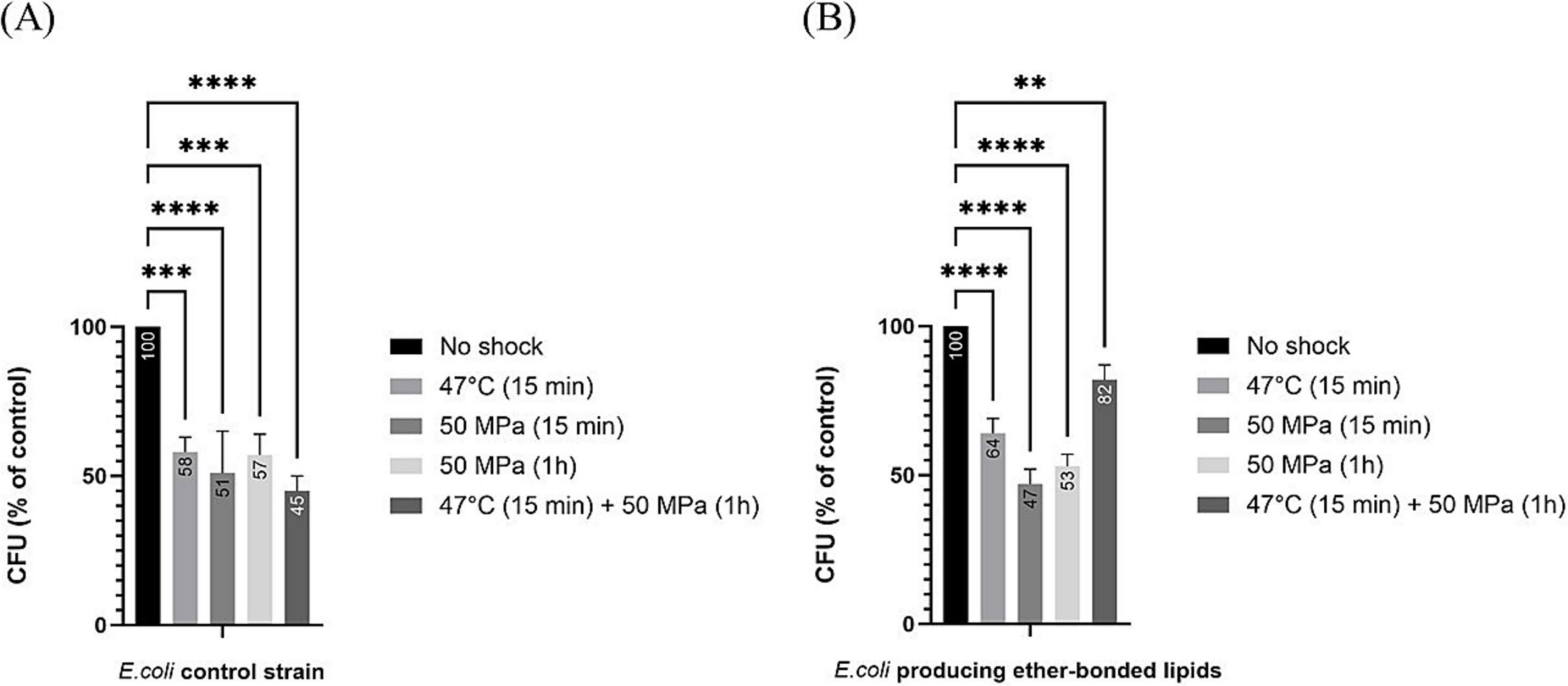
Relative colony forming unit (CFU) count after different shock treatments in *E. coli* in relation to unshocked cultures. Numbers in the bars represent the relative CFU count after shock in comparison to the unshocked cultures. **(A)** for our control strain, **(B)** for *E. coli* producing bacterial ether-bonded membrane lipids - All experiments were performed in triplicate, and the error bars indicate ± SD. ** means significant (*p* ≤ 0.01), *** and **** means strongly significant (*p* ≤ 0.001 and *p* ≤ 0.0001 respectively).

The strain ‘*E. coli* bacterial ether-based lipids’ was treated as specified above and then plated to estimate the robustness or CFU grown after shock (Figure 1), as well as percentage of CFU grown after shock with respect to the unshocked control (Figure 2). The robustness results as CFUs grown on plate after shock, showed no differences in CFUs between the control *E. coli* strain not producing ether-bonded lipids and the one producing them, being both non-shocked (i.e., growth at 37°C), indicating that producing those lipids does not affect the robustness in optimal conditions (Figure 1). Short shocks (15 min) treatments of HHP (50 MPa) or high temperature (47°C) successfully reduced the robustness of both *E. coli* producing bacterial ether-based lipids and its control strain. However, for each shock treatment of either HHP or temperature, no significant difference of robustness was observed between *E. coli* producing bacterial ether lipids and its control strain. Lack of difference could either indicate that (i) the production of bacterial ether-bonded lipids by *E. coli* does not facilitate the recovery after shock, (ii) the contribution of these ether-bonded lipids to the membrane is not enough to see an effect, possibly due to its low relative abundance within the membrane, or (iii) the shocking time is not enough to see an effect. To assess the last option, we extended the incubation time to 1 h under HHP to allow more time for the effects of the shock to affect the cells.

Notable differences in robustness, measured as CFUs grown on a plate after shock, became evident when the strains were exposed to both HHP and higher temperatures (i.e., 47°C), as in such cases, the presence of ether-bonds was beneficial with robustness increased at atmospheric pressure (6% increase in CFUs compared to those of control cells not producing ether-bonded lipids), and significantly boosted when HHP 50 MPa was applied together with the increase of temperature to 47°C (37% increase in CFUs compared to control not producing ether-bonds) (Figure 2). This confirms that the shock’s duration is important to see the effect, and it also confirms that the presence of ether-bonded lipids in the membrane is beneficial for overcoming a situation of thermal stress applied together with HHP. It is worth highlighting that the improvement on the survival or increase in robustness observed in this strain was only significant when both environmental factors were applied at the same time. The synergistic effect of multiple environmental factors has been previously observed. Indeed, such an effect has been observed in food preservation when salt gave protection against acid treatment when both factors are applied together (Lee and Kang, 2009). Cold temperature and HHP typically induce the same membrane adaptation. Consequently, combining both high temperature and HHP amplifies the challenges to maintain homeoviscosity (Winter et al., 2005). Short shocks (15 min) treatments of HHP (50 MPa) or high temperature (47°C) successfully reduced the robustness of both *E. coli* producing bacterial ether-based lipids and its control strain. However, for each shock treatment of either HHP or temperature, no significant difference of robustness was observed between *E. coli* producing bacterial ether lipids and its control strain.

To compare the effect of the different shocks in comparison to the robustness of unshocked *E. coli* (either producing or not ether-bonded bacterial membrane lipids), we also analyzed the data as percentage of CFU grown after shock with respect to the CFUs grown of the respective *E. coli* strain unshocked (Figure 2). Data indicated that all shock treatments led to a notable reduction in CFU counts with respect to the unshocked controls. In the control strain not producing the bacterial ether-bonded membrane lipids, the shock treatment resulted in a reduction of CFU of about 50% (Figure 2A). A similar result was observed for the unshocked *E. coli* producing ether-bonded membrane lipids (Figure 2B).

### 3.4 Effect of the presence of ether-bonded archaeal membrane lipids on robustness

To assess the effect of archaeal isoprenoid-based ether-bonded membrane lipids, here referred to archaetidylglycerol (AG), in the membrane of *E. coli* mostly consisting of fatty acid ester-bonded lipids (Supplementary Figure S2), the same shock treatments were applied as described above.

**Figure 3:**
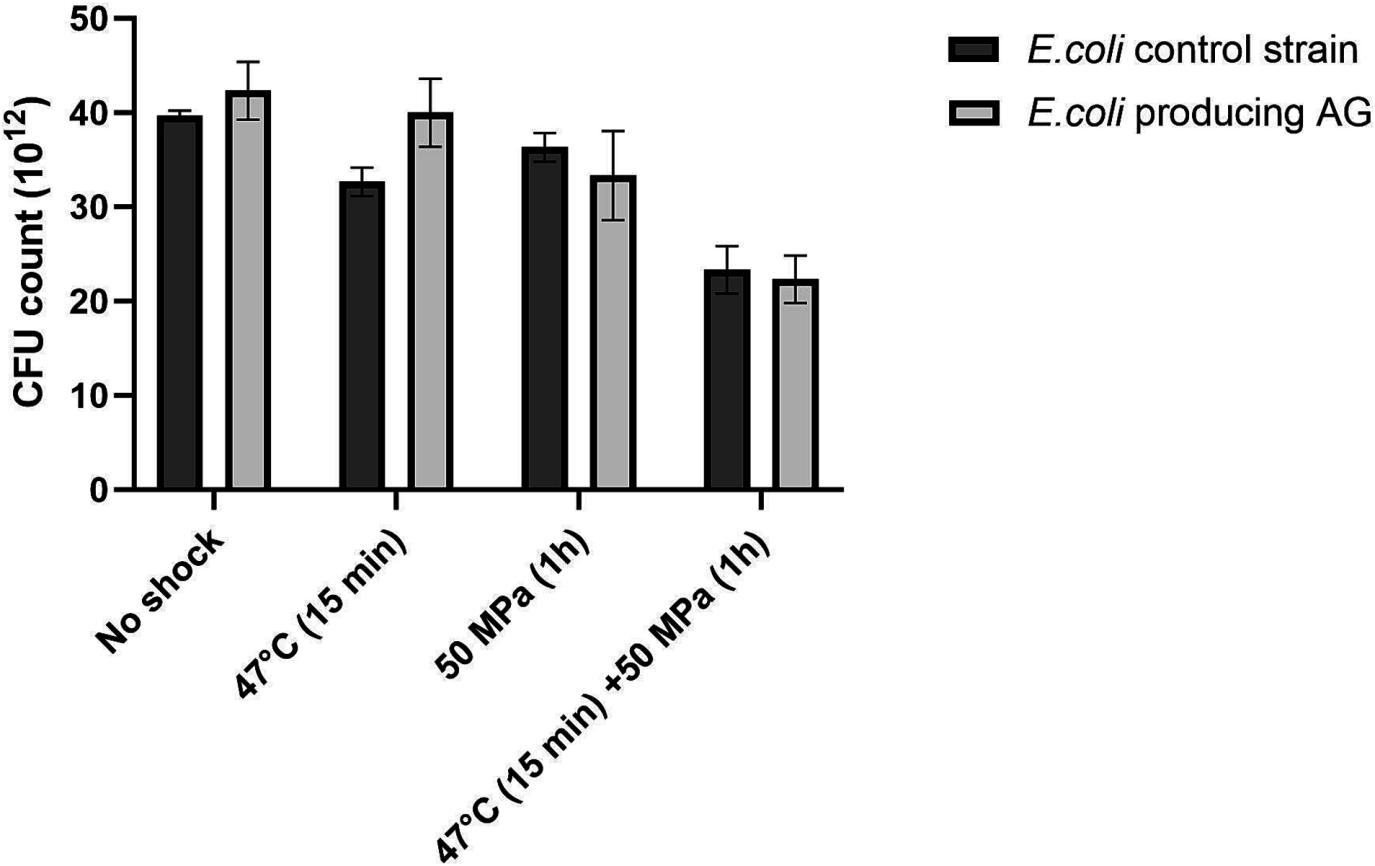
Colony forming units (CFU) absolute count in *E. coli* control strain and *E. coli* producing archaeal ether-bonded lipids (archaetidylglycerol, AG), after different shock treatment - All experiments were performed in triplicate, and the error bars indicate ± SD.

The presence of AG in *E. coli* had no discernible impact on the robustness (as CFU grown on a plate after shock) in any of the tested conditions, compared to the control strain not producing the AG (Figure 3), which was not the case for the *E. coli* strain harboring bacterial ether-bonded lipids showing a higher robustness in comparison with the control strain when subjected to higher temperature and HHP (Figure 1).

**Figure 4:**
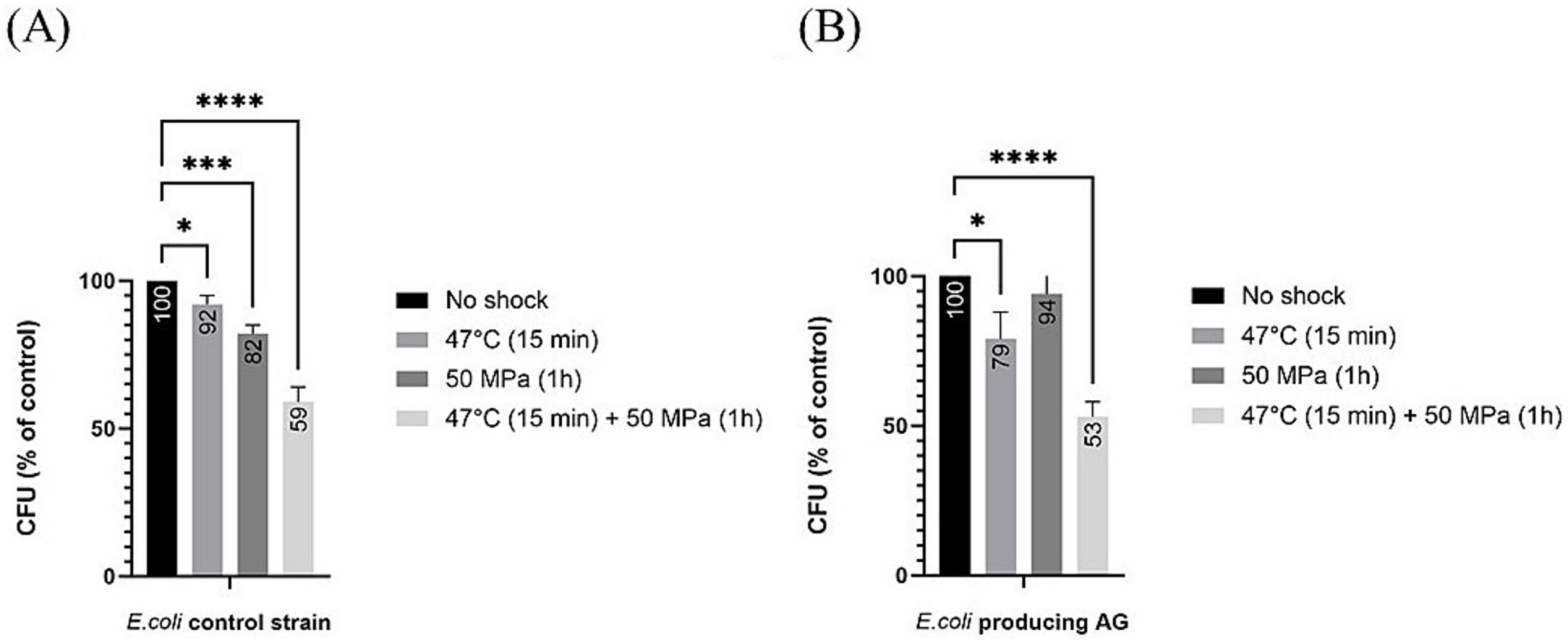
Relative colony forming unit (CFU) count after different shock treatments in *E. coli* producing archaeal ether-bonded membrane lipids in relation to unshocked cultures **(A)** for our control strain **(B)** for *E. coli* producing archaeal ether-bonded lipid*s*. All experiments were performed in triplicate, and the error bars indicate ± SD. * means slightly significant (*p* ≤ 0.05), ** means significant (*p* ≤ 0.01), *** and **** means strongly significant (*p* ≤ 0.001 and *p* ≤ 0.0001 respectively).

Regarding estimations of robustness as percentage of CFU grown after shock with respect to the unshocked control, for the control strain not producing AG, all treatments led to a significant decrease in CFUs (Figure 4), which shows that the shocks were challenging for the host, as seen before for the *E. coli* strain expressing bacterial ether-bonded membrane lipids (Figure 2). This decrease in the robustness with respect to the unshocked *E. coli* strain was steady with increasing stress conditions, with an 8% decrease with high temperature treatment at atmospheric pressure, 18% decrease at 50 MPa and optimal growth temperature, and 41% decrease at the 50 MPa and 47°C shock treatment for the *E. coli* control strains not producing AG (Figure 4A). For the *E. coli* producing AG, all applied stress conditions induced a significant decrease in robustness (% CFU with respect to unshocked) with exception from the treatment at HHP 50 MPa at optimal temperature (Figure 4B). At atmospheric pressure and 47°C, the decrease was 21%, while at 50 MPa and 47°C, the decrease was 47% (Figure 4B). These results suggest that temperature, more than pressure affect the robustness of *E. coli* producing AG. At atmospheric pressure, despite longer exposure to 47°C (15 min performed in the current study vs. 2 min performed by Caforio et al. (2018)), a similar decrease of CFU was observed, suggesting that the presence of archaeal lipids in this strain did not further impair the heat-shock response of *E. coli*.

### 3.5 Morphological impact of HHP and temperature on ether-bonded membrane lipids in the membrane

**Figure 5:**
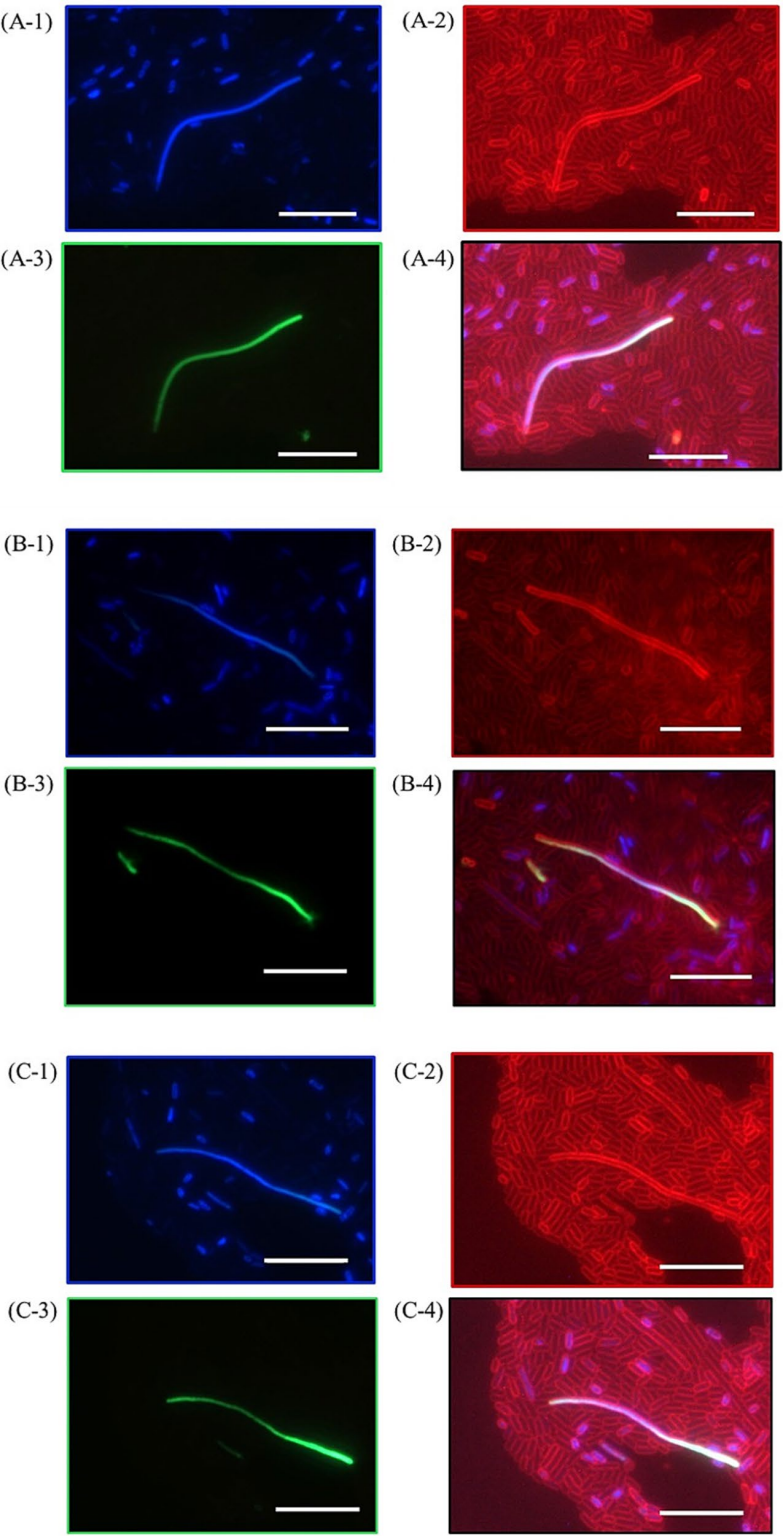
Pictures of *E. coli* producing bacterial ether-bonded lipids after a pressure shock for 47°C for 15 min and 50 MPa for 1 h visualized with fluorescent microscopy after staining, showing three occurrences of distorted cells **(A–C)**. **(A-1)**, **(B-1)**, and **(C-1)** show DNA stains through DAPI (blue, membrane permeable), **(A-2)**, **(B-2)** and **(C-2)** show membrane stain FM4-64 (red), **(A-3)**, **(B-3)** and **(C-3)** show DNA stains through SYTOX Green (membrane impermeable) and **(A-4)**, **(B-4)** and **(C-4)** show merged picture combining FM4-64, DAPI and SYTOX Green. White bars represent a 10 μm scale bar.

Further investigation into *E. coli* cultures was conducted, assessing the morphological differences between shocked and unshocked cultures. Our findings indicated the presence of bacterial ether-based lipids in the lipid membrane boosted robustness against combined high temperature and HHP treatments, whereas the presence of archaeal ether lipids (AG) maintained *E. coli’s* robustness (Figures 1, 3). However, both *E. coli* strains exhibited considerable morphological variability following all shock treatments, indicating significant stress on the organisms upon high temperature and/or HHP shock (Figure 5). To examine the effect of HHP and high temperature treatment on the morphology of the cells either containing bacterial ether-bonded membrane lipids or archaeal ones (i.e., AG), a combination of fluorescent dyes; SYTOX–green (membrane impermeable dye) and DAPI (membrane permeable dye) were employed to stain nucleic acids, and FM4-64 (a lipophilic stain) was used to dye the cell membrane. As a result, cells with an intact membrane showed as blue (Figures 5A-1, B-1, C-1) and cells with compromised membrane showed a fluorescent green signal (Figures 5A-3, B-3, C-3), both with a red colored membrane (Figures 5A-2, B-2, C-2). Upon microscopy analyses of cell morphology, some differences were observed between shocked cultures and unshocked cultures of both *E. coli* under study. Under the microscope, cultures of *E. coli* exhibited a similar rod-shaped morphology at atmospheric pressure, regardless of their lipid composition (i.e., *E. coli* harboring bacterial ether-bonded or archaeal ether-bonded membrane lipids). However, elongated cells were observed occasionally after the 47°C and 50 MPa shock treatment (Figure 5). Distorted cells presented a green-to-blue gradient due to simultaneous DAPI (blue) and SYTOX green staining on their nucleic acids (Figures 5A-4, B-4, C-4). The occurrence of those elongated cells (>20 μm) was too low to establish a representative ratio of the population. Moreover, the microscopy visualization was delayed overnight, potentially allowing for normal cellular division to resume during this interval, and thereby potentially accentuating the observed morphological variability. Visualization immediately after incubation would likely provide insight on the effect of pressurization and decompression on cell morphology, potentially display more elongated cells. In addition, the difference of cell size could be due to cell fusion, or cell elongation and our set-up does not allow to differentiate the two. These morphological changes were not observed in any other cultures, suggesting that they are indeed induced by the high pressure and high temperature treatment. Their absence from other shock treatment emphasizes the importance of studying the synergistic effect of pressure and temperature rather than those two parameters separately. Mañas and Mackey (2004), reported cell cultures react different according to their growth-phase. In addition, they found that for *E. coli* cells, membranes in exponential-phase cells are more pressure sensitive than those in the stationary phase. They also reported fluorescent area of condensed RNA caused by pressurization in cells in the stationary phase, which could explain the gradient we observed in our study. As SYTOX Green is an impermeable membrane dye, if observed inside of a cell suggests that the membrane is compromised allowing the dye to get into the cell. The gradient observed in Figures 5A-4, B-4, C-4 by the staining SYTOX green could thus show the path through which it diffused through the cells, with a more intense green glow on one tip of the cell, suggesting the membrane was compromised. This difference in the staining of SYTOX green could indicate where the cell was damaged, particularly so as all other cells were stained uniformly.

**Figure 6:**
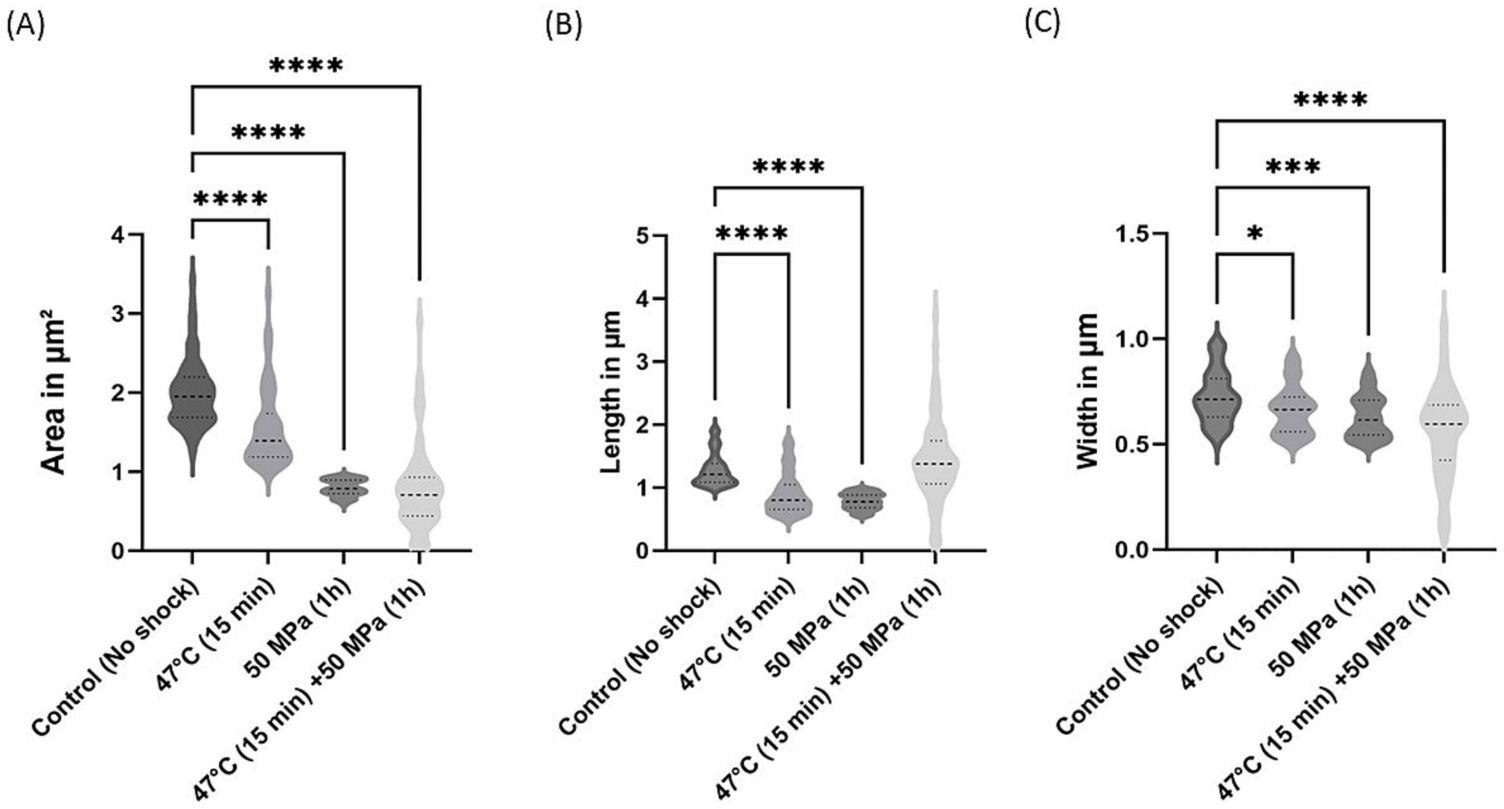
Morphological changes in *E. coli* with bacterial ether-bonded lipids after shock treatments. **(A)** Area variations, **(B)** Length variations and **(C)** Width variations. The error bars indicate ± SD. * means slightly significant (*p* ≤ 0.05), ** means significant (*p* ≤ 0.01), *** and **** means strongly significant (*p* ≤ 0.001 and *p* ≤ 0.0001 respectively).

**Figure 7:**
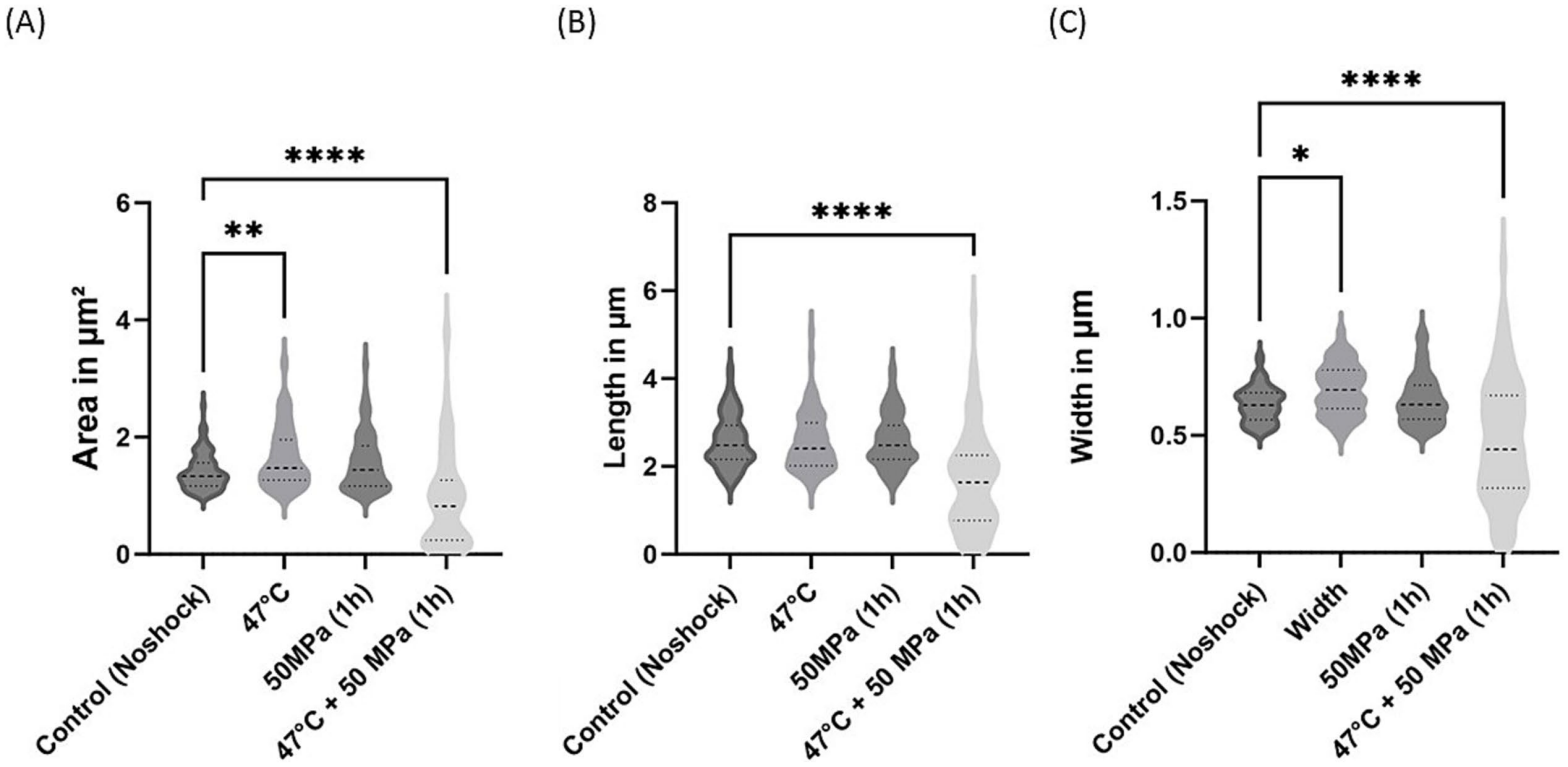
Morphological changes in *E. coli* synthesizing archaeal ether-bonded lipids (i.e., archaetidylglycerol, AG) after shock treatments. **(A)** Area variations, **(B)** length variations, and **(C)** width variations. The error bars indicate ± SD. * means slightly significant (*p* ≤ 0.05), ** means significant (*p* ≤ 0.01), *** and **** means strongly significant (*p* ≤ 0.001 and *p* ≤ 0.0001 respectively).

Further investigation into *E. coli* morphology was conducted, assessing the morphological differences between shocked and unshocked cultures (Figures 6, 7). Both *E. coli* strains exhibited considerable morphological variability following shock treatment. In *E. coli* containing bacterial ether-bonded membrane lipids, we noted a reduction in cell area under increased pressure (Figure 6A). Additionally, a decrease in cell length was observed at 47°C and 50 MPa (Figure 6B), although no significant change in length was evident during the combined shock of high temperature and HHP (Figure 6B). However, the notable variability in length and width observed under the combined shock conditions makes comparisons with other treatments difficult. This variability may be due to stochastic effects attributed to size fluctuations in impaired cell divisions when both treatments are applied at the same time. Notably, width emerged as the most consistent parameter across both strains, suggesting that while cells were not elongated by HHP, their division was hindered (Figures 6C, 7C; Kutalik et al., 2005).

For the *E. coli* strain producing archaeal ether-bonded membrane lipids (i.e., AG), notable morphological changes were evident under the combined shock treatment of high temperature and high pressure, manifested by reductions in cell area, length, and width (Figure 7). However, the substantial variability in these parameters complicates definitive conclusions. Specifically, a significant decrease in cell area was observed under HHP conditions, accompanied by reduced length following shock treatments (Figures 7A,B). Nevertheless, cell width remained consistent across all shock treatments, except in the combined shocks treatment (Figure 7C). Caforio et al. conducted similar morphological analysis of this strain and observed comparable length when *E. coli* producing AG was induced with low concentrations of IPTG (<10 μM). However, at high concentrations (>100 μM), elongated cells and distorted cells with appendages were observed (Caforio et al., 2018). In the current study, cells were induced with a much higher concentrations (5 mM), however we did not observe analogous deformities.

To fully assess the potential of hybrid membranes, longer incubation times and further molecular analysis, such as proteomics, would provide insight on adaptive strategies at play. Furthermore, being able to visualize cells without decompression would elucidate morphological changes induced by HHP.

## 4 Conclusion

While the current study does not specifically identify the role that ether bonds play in increased survival rate at high temperature and high pressure, our results show that it is crucial to consider those two parameters together when considering environmentally relevant conditions affecting the survival of microorganisms through the maintenance of their membrane stability (Xiao et al., 2021). Indeed, while the robustness of *E. coli* with bacterial ether bonds was slightly increased at high temperature and atmospheric pressure, it remained insignificant. However, a significant increase of robustness arose at high pressure and high temperature. This suggests that microorganisms harboring both bacterial ester and ether-bonds lipids might have a wider range of thermal and HHP adaptation. Considering that early evolution likely happened in the marine environment, temperature and HHP are legitimate parameters to consider when studying the driving forces of the lipid divide. In addition, the relative abundance of ether-bonded lipids can lead to different results as the ones reported here.

Data derived from our study proves it is crucial to consider the lipid membrane and survival effects of the application of more than one abiotic factor (i.e., a combination of high temperature and pressure), which was often overlooked in previous studies. Future studies in model microorganisms synthesizing membranes with mixed characteristics in different proportions will further clarify the driving forces of cellular membrane evolution in both the origin of life and of eukaryotic cells.

## Data Availability

The datasets presented in this study can be found in online repositories. The names of the repository/repositories and accession number(s) can be found at: https://zenodo.org/records/12818159, https://zenodo.org/records/12817938.

## References

[ref1] Bale N. J. Ding S. Hopmans E. C. Arts M. G. I. Villanueva L. Boschman C.. (2021). Lipidomics of environmental microbial communities. I: visualization of component distributions using untargeted analysis of high-resolution mass spectrometry data. Front. Microbiol. 12:659302. doi: 10.3389/fmicb.2021.659302, PMID: 34367080 PMC8343106

[ref2] Bartlett D. H. (2002). Pressure effects on in vivo microbial processes. BBA-Protein Struct. M. 1595, 367–381. doi: 10.1016/S0167-4838(01)00357-0 11983409

[ref3] Caforio A. Siliakus M. F. Exterkate M. Jain S. Jumde V. R. Andringa R. L. H.. (2018). Converting *Escherichia coli* into an archaebacterium with a hybrid heterochiral membrane. Proc. Natl. Acad. Sci. USA 115, 3704–3709. doi: 10.1073/pnas.1721604115, PMID: 29555770 PMC5889666

[ref4] Daniel I. Oger P. Winter R. (2006). Origins of life and biochemistry under high-pressure conditions. Chem. Soc. Rev. 35, 858–875. doi: 10.1039/b517766a, PMID: 17003893

[ref5] Halamka T. A. Raberg J. H. McFarlin J. M. Younkin A. D. Mulligan C. Liu X. L.. (2023). Production of diverse brGDGTs by Acidobacterium Solibacter usitatus in response to temperature, pH, and O2 provides a culturing perspective on brGDGT proxies and biosynthesis. Geobiology 21, 102–118. doi: 10.1111/gbi.12525 36150122 PMC10087280

[ref6] Jain S. Caforio A. Driessen A. J. M. (2014). Biosynthesis of archaeal membrane ether lipids. Front. Microbiol. 5:641. doi: 10.3389/fmicb.2014.00641, PMID: 25505460 PMC4244643

[ref7] Koga Y. (2011). Early evolution of membrane lipids: how did the lipid divide occur? J. Mol. Evol. 72, 274–282. doi: 10.1007/s00239-011-9428-5, PMID: 21259003

[ref8] Koga Y. Morii H. (2005). Recent advances in structural research on ether lipids from archaea including comparative and physiological aspects. Biosci. Biotechnol. Biochem. 69, 2019–2034. doi: 10.1271/bbb.69.2019, PMID: 16306681

[ref9] Kutalik Z. Razaz M. Elfwing A. Ballagi A. Baranyi J. (2005). Stochastic modelling of individual cell growth using flow chamber microscopy images. Int. J. Food Microbiol. 105, 177–190. doi: 10.1016/j.ijfoodmicro.2005.04.026 16091296

[ref10] Lee S. Y. Kang D. H. (2009). Combined effects of heat, acetic acid, and salt for inactivating *Escherichia coli* O157: H7 in laboratory media. Food Control 20, 1006–1012. doi: 10.1016/j.foodcont.2008.12.002

[ref11] Lehmann M. Prohaska C. Zeldes B. Poehlein A. Daniel R. Basen M. (2023). Adaptive laboratory evolution of a thermophile toward a reduced growth temperature optimum. Front. Microbiol. 14:1265216. doi: 10.3389/fmicb.2023.1265216, PMID: 37901835 PMC10601643

[ref12] Mañas P. Mackey B. M. (2004). Morphological and physiological changes induced by high hydrostatic pressure in exponential-and stationary-phase cells of *Escherichia coli*: relationship with cell death. Appl. Environ. Micro. 70, 1545–1554. doi: 10.1128/aem.70.3.1545-1554.2004 PMC368324 15006777

[ref13] Marietou A. Nguyen A. T. T. Allen E. E. Bartlett D. H. (2014). Adaptive laboratory evolution of *Escherichia coli* K-12 MG1655 for growth at high hydrostatic pressure. Front. Microbiol. 5:749. doi: 10.3389/fmicb.2014.00749, PMID: 25610434 PMC4285802

[ref14] Sahonero-Canavesi D. X. Siliakus M. F. Asbun A. A. Koenen M. Von Meijenfeldt F. A. B. Boeren S.. (2022a). Disentangling the lipid divide: identification of key enzymes for the biosynthesis of membrane-spanning and ether lipids in Bacteria. Sci. Adv. 8:eabq8652. doi: 10.1126/sciadv.abq8652, PMID: 36525503 PMC13159169

[ref15] Sahonero-Canavesi D. X. Villanueva L. Bale N. J. Bosviel J. Koenen M. Hopmans E. C.. (2022b). Changes in the distribution of membrane lipids during growth of *Thermotoga maritima* at different temperatures: Indications for the potential mechanism of biosynthesis of ether-bound diabolic acid (membrane-spanning) lipids. Available online at: https://journals.asm.org/journal/aem 10.1128/AEM.01763-21 PMC8788747 34731048

[ref16] Schouten S. Hopmans E. C. Damsté J. S. S. (2013). Organic geochemistry the organic geochemistry of glycerol dialkyl glycerol tetraether lipids: a review. Org. Geochem. 54, 19–61. doi: 10.1016/j.orggeochem.2012.09.006

[ref17] Siliakus M. F. van der Oost J. Kengen S. W. M. (2017). Adaptations of archaeal and bacterial membranes to variations in temperature, pH and pressure. Extremophiles 21, 651–670. doi: 10.1007/s00792-017-0939-x, PMID: 28508135 PMC5487899

[ref18] Tamby A. Sinninghe Damsté J. S. Villanueva L. (2023). Microbial membrane lipid adaptations to high hydrostatic pressure in the marine environment. Front. Mol. Biosci. 9:1058381. doi: 10.3389/fmolb.2022.1058381, PMID: 36685280 PMC9853057

[ref19] Valentine D. L. (2007). Adaptations to energy stress dictate the ecology and evolution of the Archaea. Nat Rev Microbiol 5, 316–323. doi: 10.1038/nrmicro1619 17334387

[ref20] Villanueva L. Schouten S. Damsté J. S. S. (2017). Phylogenomic analysis of lipid biosynthetic genes of Archaea shed light on the ‘lipid divide. Environ. Microbiol. 19, 54–69. doi: 10.1111/1462-2920.13361 27112361

[ref21] Villanueva L. Von Meijenfeldt F. A. B. Westbye A. B. Hopmans E. C. Dutilh B. E. Damsté J. S. S. (2020). Bridging the membrane lipid divide: bacteria of the FCB group superphylum have the potential to synthesize archaeal ether lipids. ISME J. 15, 168–182. doi: 10.1038/s41396-020-00772-2, PMID: 32929208 PMC7852524

[ref22] Winnikoff J. R. Milshteyn D. Vargas-Urbano S. J. Pedraza-Joya M. A. Armando A. M. Quehenberger O.. (2024). Homeocurvature adaptation of phospholipids to pressure in deep-sea invertebrates. Sciences 384, 1482–1488. doi: 10.1126/science.adm7607, PMID: 38935710 PMC11593575

[ref23] Winter R. Dzwolak W. Wolynes P. G. Dobson C. M. Saykally R. J. (2005). Exploring the temperature-pressure configurational landscape of biomolecules: from lipid membranes to proteins. Philos. Trans. R. Soc. A Math. Phys. Eng. Sci. 363, 537–563. doi: 10.1098/rsta.2004.1507, PMID: 15664898

[ref24] Xiao X. Zhang Y. Wang F. (2021). Hydrostatic pressure is the universal key driver of microbial evolution in the deep ocean and beyond. Environ. Microbiol. Rep. 13, 68–72. doi: 10.1111/1758-2229.12915, PMID: 33398931

[ref25] Yadav S. Villanueva L. Bale N. Koenen M. Hopmans E. C. Damsté J. S. S. (2020). Physiological, chemotaxonomic and genomic characterization of two novel piezotolerant bacteria of the family Marinifilaceae isolated from sulfidic waters of the Black Sea. Syst. Appl. Microbiol. 43:126122. doi: 10.1016/j.syapm.2020.126122, PMID: 32847788

[ref26] Yeagle P. L. (1989). Lipid regulation of cell membrane structure and function. FASEB J. 3, 1833–1842. doi: 10.1096/fasebj.3.7.2469614 2469614

[ref27] Zapparoli G. (2004). Colony dimorphism associated with stress resistance in *Oenococcus oeni* VP01 cells during stationary growth phase. FEMS Microbiol. Lett. 239, 261–265. doi: 10.1016/j.femsle.2004.08.047, PMID: 15476975

