# Supplementary material for: Exploring robustness of hybrid membranes under high hydrostatic pressure and temperature
Source: Front Microbiol. 2024 Nov 14;15:1470844. doi: 10.3389/fmicb.2024.1470844 (PMC11604127; doi:10.3389/fmicb.2024.1470844)

***Supplementary Material***

Supplementary Figure 1. (A) Partial GC extracted ion chromatograms (m/z 205; 24.75 – 25.75 min) of the extract of *E.coli* control strain (top) and *E.coli* producing ether lipids (bottom), (B) Mass spectra of the C16 glycerol monoether (1-MGE)


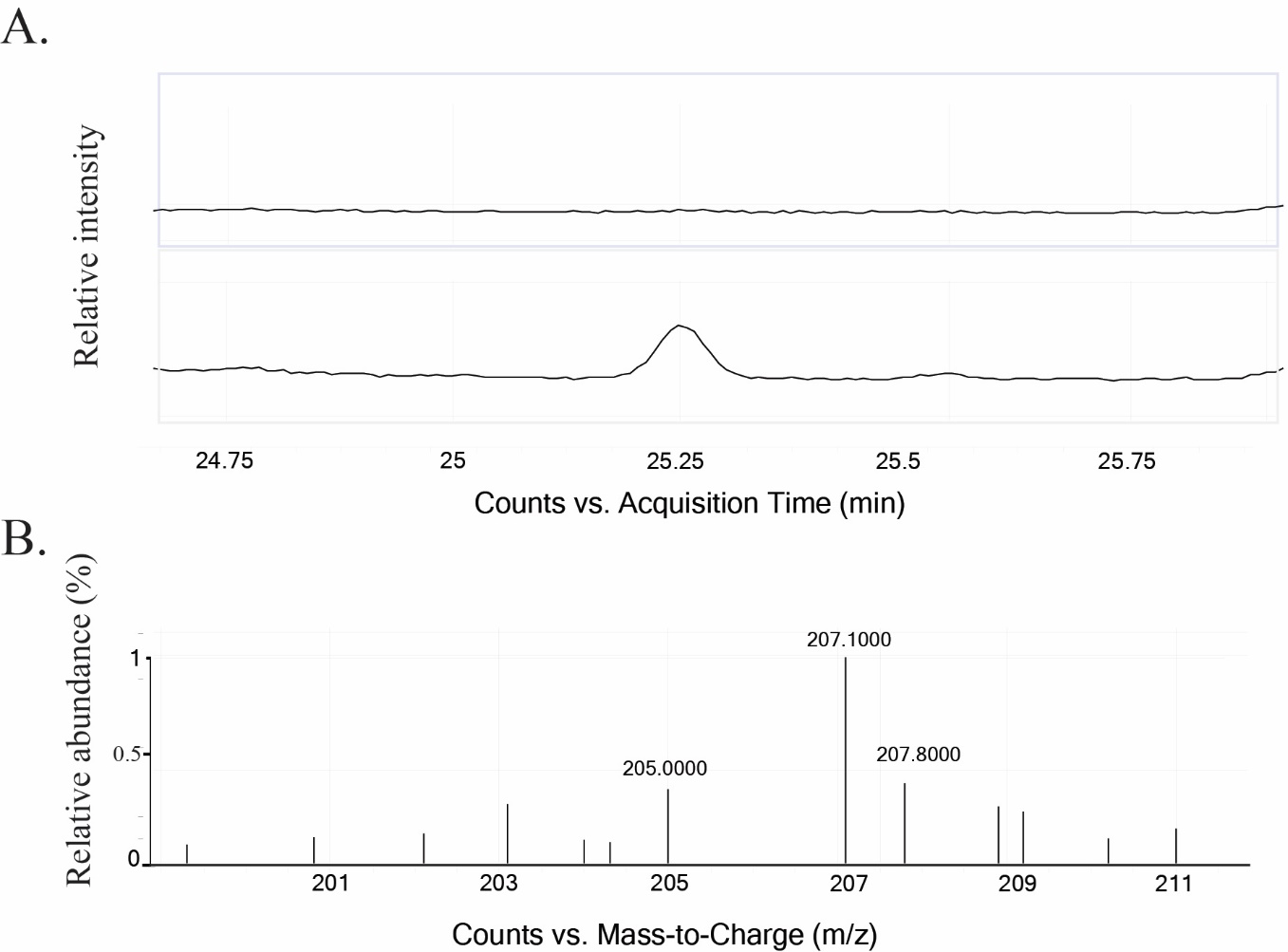


Supplementary Figure 2. Partial base peak chromatogram of UHPLC-HRMS analysis of IPLs (m/z 788-800; t=12-19 min) of the extract of *E.coli* producing AG (m/z = 791.5585, 808.5881, 813.5404). (A) represents the ms spectra. Top part represents the full ms spectra. Middle the base peak of 791.5585, 808.5881 and 813.5404. Bottom represents the ms2 of 808.588. (B) represents the ESI full ms between with mass range between 788 and 820


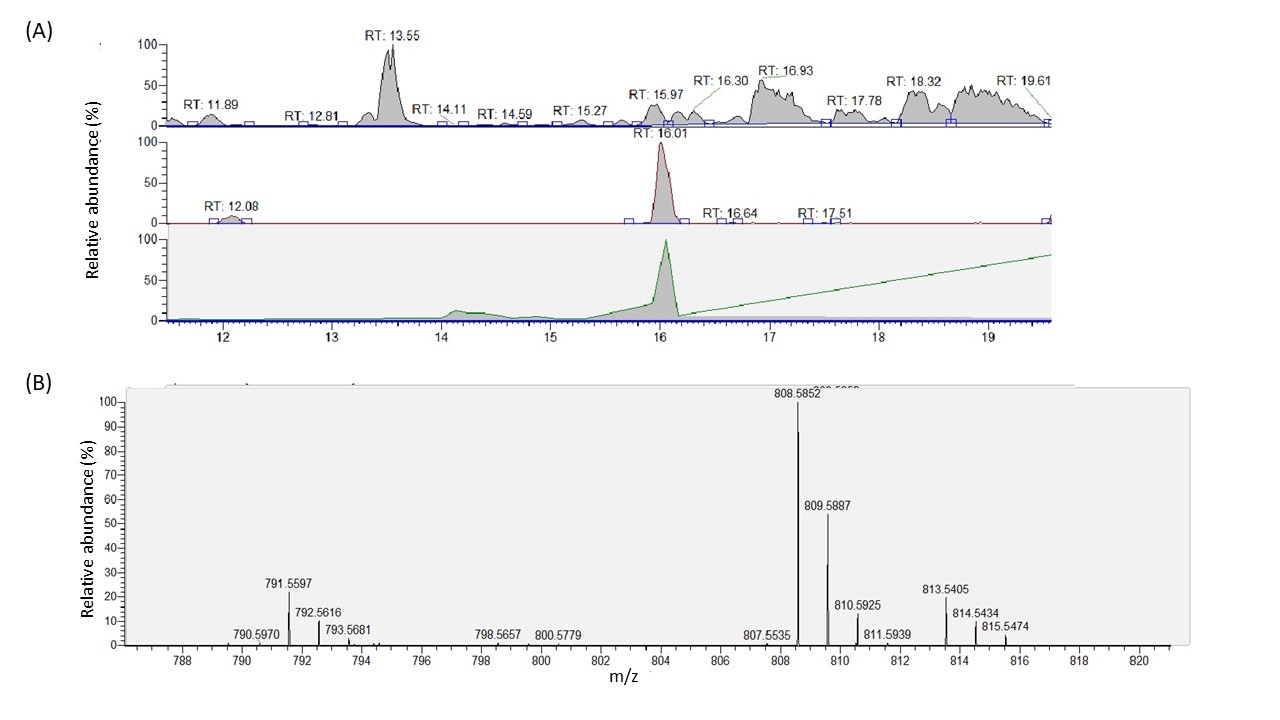

Supplement: Supplementary file 1 [file Supplementary_file_1.docx]
